# Chimeric Antigen Receptor (CAR) T-cell Therapy in the Treatment of Diffuse Large B-cell Lymphoma (DLBCL): A Systematic Review

**DOI:** 10.7759/cureus.75854

**Published:** 2024-12-17

**Authors:** Amir T Ibrahiam, Sunitha Geddada, Najeeb Ullah, Zahraa m Al-Qassab, Osman Ahmed, Safeera Khan

**Affiliations:** 1 Internal Medicine, California Institute of Behavioral Neurosciences and Psychology, Fairfield, USA; 2 General Surgery, California Institute of Behavioral Neurosciences and Psychology, Fairfield, USA

**Keywords:** car t-cell therapy, complete response, dlbcl, efficacy, overall survival, side effects

## Abstract

Chimeric antigen receptor (CAR) T-cell therapy has shown very promising results in the treatment of refractory or relapsed diffuse large B-cell lymphoma (DLBCL). This systematic review evaluates the effectiveness and side effects of CAR T-cell therapies, focusing on factors affecting both clinical outcomes and adverse effects. This review included data from 14 studies involving 1392 patients with DLBCL who underwent CAR T-cell therapy. These studies include both randomized clinical trials and observational studies, which would help to analyze the effectiveness and safety profiles. The review highlights that CAR T-cell therapies, mainly tisagenlecleucel (Tisa-cel) and axicabtagene ciloleucel (Axi-cel), have shown superior effectiveness in comparison to standard chemotherapy in patients with relapsed or refractory DLBCL. Lisocabtagene maraleucel (Liso-cel) showed significant improvement outcomes in event-free and progression-free survival. However, CAR T-cell therapies are associated with many side effects. The most common side effects include hematologic toxicity, prolonged neutropenia, and infections, while clinical outcomes are highly impacted by many factors, which include a pro-inflammatory state, PPM1D gene mutation, infusion timing, and circulating monocytes.

## Introduction and background

“There is far more danger of harm than there is hope of good in any radical changes," said Calvin Coolidge.

Diffuse large B-cell lymphoma (DLBCL) is among the commonest types of non-Hodgkin lymphoma (NHL), characterized by rapidly growing tumors in the reticuloendothelial system or other tissues and organs. The overall survival rate is 64.4% at five years from the onset of diagnosis, although this may vary based on the cancer stage. While first-line treatments, such as the cyclophosphamide, doxorubicin, vincristine, and prednisone (R-CHOP) regimen, achieve remission in only 30-40% of cases, patients may relapse or be refractory to treatment, creating a great challenge in treating those patients [1].

Chimeric antigen receptor (CAR) T-cell therapy represents a remarkable advance in the treatment of relapsed or refractory diffuse large-cell lymphoma. Unlike standard chemotherapy, CAR T-cell therapy causes genetic modification of a patient's T-cells to express a receptor that specifically targets the CD19 antigen on B-cells, leading to their destruction [2].

While many studies have focused on either the effectiveness or the side effects of CAR T-cell therapy, our aim is to explore both the benefits and risks of this new treatment modality. This approach could help shape the future of CAR T-cell infusion therapy for DLBCL. Since 2017, several CAR T-cell treatments have been approved by the Food and Drug Administration (FDA), with tisagenlecleucel (Tisa-cel) and axicabtagene ciloleucel (Axi-cel) as the most common treatments used for relapsed/refractory DLBCL in children and adults, respectively, particularly in patients who have failed or relapsed after multiple cycles of therapies. However, CAR T-cell therapy has its own risks, as its use is associated with severe toxicities, such as cytokine release syndrome (CRS) and neurotoxicity, which require careful clinical judgment and treatment [3].

While several studies have shown the efficacy of CAR T-cell therapy in achieving complete response rates in patients with DLBCL, there are notable variations in outcomes based on patient and tumor characteristics. This systematic review focuses on evaluating the overall effectiveness and side effects of CAR T-cell therapy in the treatment of relapsed or refractory DLBCL, with a focus on understanding the factors that affect clinical outcomes and the development of treatment-related toxicities, which include tumor microenvironment, tumor burden, circulating monocytes, single nucleotide polymorphism in CD19, PPM1D gene mutation, and delayed drug infusion [4-6].

## Review

Materials and methods

The systematic review was made according to Reporting Items for Systematic Reviews and Meta-Analyses (PRISMA) guidelines​ [7].

Search strategy

We performed a comprehensive search using PubMed, PubMed Central (PMC), and Cochrane Library databases to find the related studies. Our search plan included various combinations of the following keywords: "efficacy," "adverse effects," "DLBCL," and "CAR T-cell therapy."

("Immunotherapy, Adoptive/adverse effects"[Mesh] OR "Immunotherapy, Adoptive/mortality"[Mesh] OR "Immunotherapy, Adoptive/standards"[Mesh]) AND ("Lymphoma, Large B-Cell, Diffuse/blood"[Majr] OR "Lymphoma, Large B-Cell, Diffuse/drug therapy"[Majr] OR "Lymphoma, Large B-Cell, Diffuse/mortality"[Majr] OR "Lymphoma, Large B-Cell, Diffuse/therapy"[Majr]). Table 1 outlines the databases utilized and the number of papers identified from each one.

**Table 1:** Keywords/strategy used and the number of identified papers CAR T-cell therapy: chimeric antigen receptor T-cell therapy; DLBCL: diffuse large B-cell lymphoma; MDPI: Multidisciplinary Digital Publishing Institute; PMC: PubMed Central

Search strategy	Database used	Number of literature found
CAR T-Cell therapy AND DLBCL	MDPI	32
CAR T-CELL THERAPY effectiveness and adverse effects	Cochrane library	15
(“Immunotherapy, Adoptive/adverse effects" [Mesh] OR “Immunotherapy, Adoptive/mortality"[Mesh] OR "Immunotherapy, Adoptive/ standards"[Mesh]) AND (“Lymphoma, Large B-Cell, Diffuse/blood"[Majr] OR "Lymphoma, Large B-Cell, Diffuse/drug therapy"[Majr] OR "Lymphoma, Large B-Cell, Diffuse/mortality"[Majr] OR "Lymphoma, Large B-Cell, Diffuse/therapy"[Majr])	PubMed Mesh	71
CAR T-Cell therapy AND DLBCL. AND effectiveness and adverse effects	PubMed	23

Inclusion and exclusion criteria

We have included only articles published in the past five years, including papers written in English or if the full-text English language is available. We included research papers on humans only and articles focusing on the effectiveness and side effects of CAR T-cell therapy. Articles focusing on conventional chemotherapy for DLBCL were also not included. Additionally, the studies focused on the role of CAR T-cell therapy in treating cancers other than DLBCL.

Selection process

EndNote was used to organize the relevant articles, after which duplicates were removed. Each article's title and abstract were screened. Full texts of the finalized articles were then reviewed, and only the related ones were included. Then the inclusion and exclusion criteria were applied to finalize the relevant articles.

Quality Assessment of the Studies

The quality of the finalized articles was evaluated using appropriate quality appraisal tools. Observational studies were assessed using the Newcastle-Ottawa tool, while randomized clinical trials were evaluated using the Cochrane bias tool. Two reviewers independently screened and reviewed the articles, resolving any discrepancies through consensus. Only studies that met the quality appraisal criteria were included in the systematic review.

The Newcastle-Ottawa Scale (NOS) evaluates studies across three categories: selection (0-4 points), comparability (0-2 points), and outcome (0-3 points). with higher scores indicating better quality. The Cochrane Risk of Bias Tool assesses randomized controlled trials across several domains (e.g., randomization, blinding, attrition), categorizing each as low, high, or unclear risk of bias.

Studies are excluded based on high or unclear risk of bias in the Cochrane tool or if they score below five points on the NOS. Studies with significant methodological flaws, poor selection criteria, or inadequate follow-up were unsuitable for inclusion in the systematic review. Tables 2-3 show the results of the quality appraisal [8-34].

**Table 2:** Quality appraisal using Cochrane bias SOC: standard of care; RCT: randomized controlled trial; (+): indicates low risk; (?): indicates unclear risk; (-): indicates high risk

Study	Random sequence generation (selection bias)	Allocation concealment (selection bias)	Blinding of participants and personnel (performance bias)	Blinding of outcome assessment (detection bias)	Incomplete outcome data (attrition bias)	Selective reporting (reporting bias)	Other bias	Bias risk
Hirayama et al., 2024 [8]	-	?	?	+	-	+	?	High
Filosto et al., 2024 [9]	+	+	?	+	?	?	Marked as advertisement	Unclear
Abramson et al., 2023 [26]	+	+	?	+	-	?	?	Low
Messerli et al., 2023 [33]	?	?	?	+	+	-	?	High
Lievin et al., 2022 [34]	?	?	?	?	+	+	?	High

**Table 3:** Quality appraisal using the Newcastle-Ottawa tool MTV: metabolic tumor volume; ANC: absolute neutrophil count; (✓): indicates the criterion was met; (X): indicates the criterion was not met; (-): indicates unclear or intermediate assessment

study	Representatives of exposed cohort	Selection of non-exposure cohort	Ascertainment of exposed	Demonstration of the outcome of interest was not present at the start of the study	Comparability of cohort on the design or analysis	Assessment of outcome	As follow up long enough for out com to occur	Adequacy of follow up cohort	Points
Voltin et al., 2024 [10]	✓	X	✓	✓	-	✓	✓	-	5
Wudhikarn et al., 2020 [14]	✓	X	✓	✓	✓	✓	✓	✓	7
Seipel, K et al., 2023 [15]	✓	X	✓	✓	X	✓	✓	✓	6
Sang et al., 2020 [16]	✓	X	✓	✓	✓	✓	✓	✓	7
Seipel, K at al., 2023 [17]	✓	X	✓	✓	✓	✓	✓	✓	7
Rejeski et al., 2021 [18]	✓	X	✓	X	✓	✓	✓	✓	6
Nagle et al., 2021 [19]	✓	X	✓	✓	✓	✓	✓	✓	7
Li et al., 2024 [20]	✓	X	✓	X	-	✓	✓	✓	5
Jallouk et al., 2024 [21]	✓	✓	✓	✓	✓	✓	✓	✓	8
Iouino et al., 2022 [22]	✓	X	✓	✓	X	✓	✓	✓	6
Faramand et al., 2020 [23]	✓	X	✓	X	X	✓	✓	✓	5
Carnitic et al., 2024 [24]	✓	X	✓	✓	-	✓	✓	✓	6
Houot et al., 2023 [25]	✓	X	✓	✓	✓	✓	✓	✓	7
Zhao et al., 2023 [27]	✓	X	✓	✓	-	-	-	-	3
Penack et al., 2023 [28]	✓	X	✓	✓	-	✓	X	X	4
Wood et al., 2022 [29]	✓	✓	✓	-	-	-	-	X	3
Scholler et al., 2022 [30]	✓	X	✓	✓	-	✓	X	X	4
Ogasawara et al., 2022 [31]	✓	X	✓	X	-	✓	-	-	3
Farina et al., 2022 [32]	✓	X	✓	✓	-	✓	X	X	4

Data Collection Process

Once the articles were finalized for the systematic review, data was manually extracted and prepared for the discussion section.

Results

Study Identification and Selection

**Figure 1:**
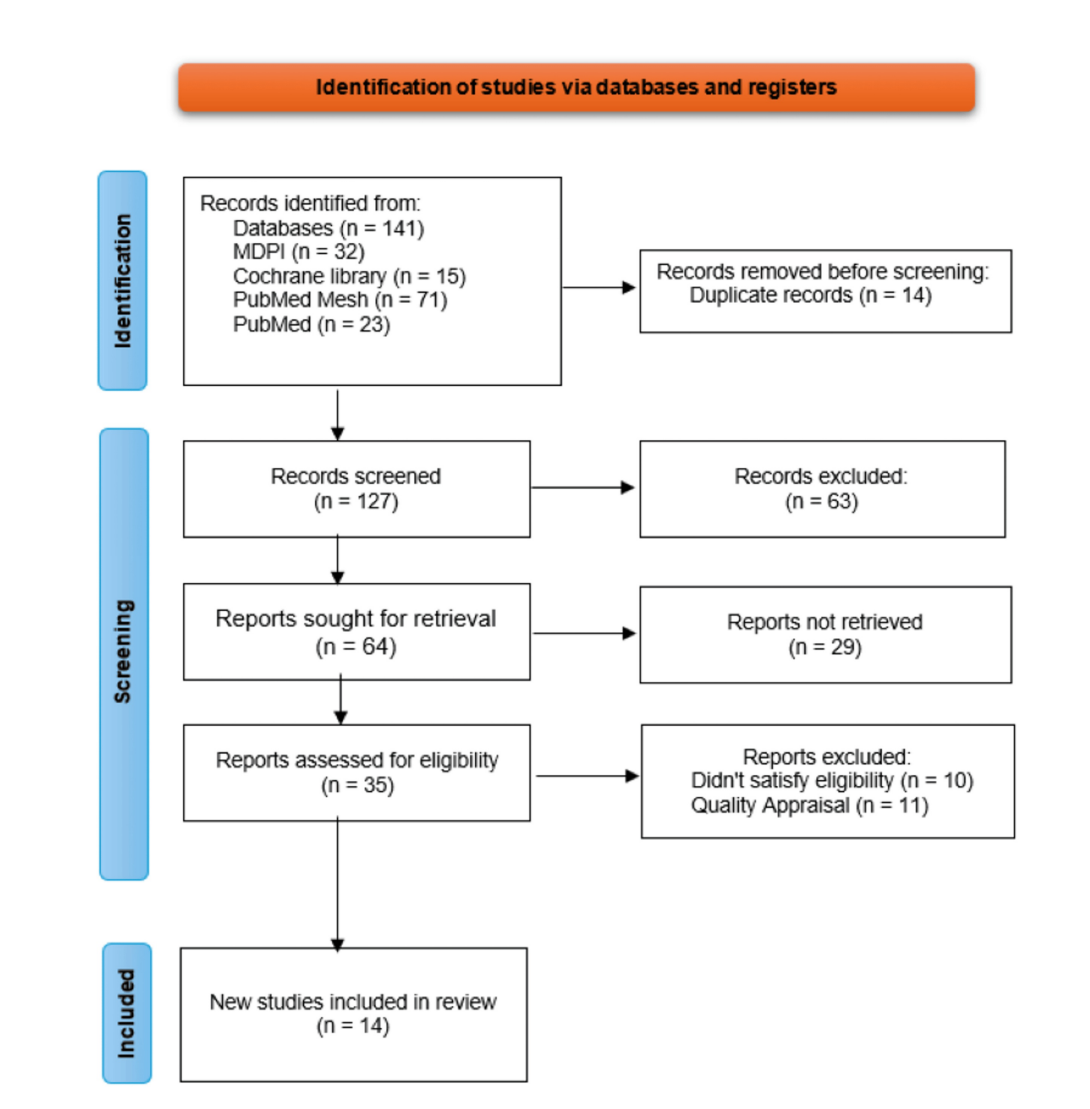
PRISMA flowchart showing the process of article selection PRISMA: Preferred Reporting Items for Systematic Reviews and Meta-Analyses;  MDPI: Multidisciplinary Digital Publishing Institute

A total of 141 relevant articles were identified across various databases. After removing 14 duplicates, the remaining articles were screened based on their titles, abstracts, and full texts. From this screening process, 35 articles were initially shortlisted. After a detailed assessment of their eligibility and quality, 14 articles were finalized for inclusion in the review [10,14-26]. The study selection process is illustrated in Figure 1, following the PRISMA flowchart.

Study Characteristics

We reviewed 14 research papers with a total of 1392 patients who received CAR T-cell therapy. The included patients are mostly adults 18 to 80 years old who mainly suffer from refractory or relapsed DLBCL, but there is no gender preference in selecting patients in all finalized studies. Out of these finalized studies, one randomized controlled trial [26], three non-randomized clinical trials [17,20,25], and the rest are observational studies [13-19,21-24]. All studies involved patients who received CAR T-cell therapy and followed up to monitor its effectiveness and possible adverse outcomes. Table 4 presents the number of participants and the variables identified from the finalized studies.

**Table 4:** Summary of the included studies Liso-cel: lisocabtagene maraleucel; Axi-cel: axicabtagene ciloleucel; Tisa-cel: tisagenlecleucel; CAR T-cell: chimeric antigen receptor T-cell; LBCL: large B-cell lymphoma; CD: cluster of differentiation; RCT: randomized controlled trial; IL: interleukin

Study	Drug studied	No. of patients	Type of study	Results/conclusion
Voltin et al., 2024 [10]	Tisa_cel Axi_cel	88	Observational study	The presence of multiple extra-nodal lesions and increased metabolic tumor volume (MTV) in LBCL is associated with worse outcomes following CAR T-cell therapy, and a lower progression-free survival rate.
Wudhikarn et al., 2020 [14]	Tisa_cel Axi_cel	60	Cohort observational study	A significant risk of bacterial infections, followed by viral infections, is among the most common side effects after CAR T-cell therapy.
Seipel et al., 2023 [15]	Anti-CD19 CAR T-cell therapy	85	Observational retrospective study	Clinical outcomes were worse in patients with PPM1D mutation which negatively affects overall survival and progression-free survival with only partial remission in 60% of patients
Sang et al., 2020 [16]	Anti-CD19 plus anti-CD20 CAR T-cell therapy	21	Phase || clinical trial	Combination of both treatments has a better outcome and manageable adverse effects
Seipel et al., 2023 [17]	Anti-CD19 CAR T-cell therapy	88	Observational retrospective study	Patients with the germline variant L174 of the CD19 antigen, caused by a single nucleotide polymorphism, showed better clinical outcomes, with better response rates and survival
Rejeski et al., 2021 [18]	Axi_cel Tisa_cel	258	Multicenter observational retrospective study	Profound and prolonged neutropenia are common side effects of CAR T-cell therapy
Nagle et al., 2021 [19]	Axi_cel Tisa_cel	31	Retrospective observational study	58% of patients developed prolonged hematologic toxicity with shorter survival
Li et al., 2024 [20]	Anti-CD19 CAR T-cell therapy	38	Non-randomized clinical trial	Persistent Cytopenia After CAR T-cell infusion could occur which indicates higher efficacy
Jallouk et al., 2024 [21]	Axicabtagene ciloleucel	240	Observational retrospective study	Worse outcome with delayed infusion which affects overall survival and progression-free survival
Iouino et al., 2022 [22]	Axicabtagene ciloleucel	60	Observational retrospective study	Bulky disease prior to lymphodepletion independently lead to worse outcomes, and patients with high-burden disease that did not respond to pre-CAR T-cell therapy had a very poor prognosis
Faramand et al., 2020 [23]	Axicabtagene ciloleucel	75	Observational study	Pro-inflammatory state and unfavorable tumor microenvironment especially high IL6 have a higher incidence of toxicity following CAR T-cell therapy
Carniti et al., 2024 [24]	Tisa_cel Axi _cel	95	Observational study	Higher circulating monocytes negatively affect response rate and pretreatment depletion has a better outcome
Houot et al., 2023 [25]	Axicabtagene ciloleucel	69	Non-randomized clinical trial	Axicabtagene demonstrated superior effectiveness compared to standard-of-care chemotherapy
Abramson et al., 2023 [26]	Lisocabtagene maraleucel	184	RCT, phase 3 transform study	Lisocabtagene demonstrated superior effectiveness compared to standard-of-care chemotherapy

Discussion

DLBCL is one of the most prevalent forms of NHL, usually treated with the standard CHOP regimen, which includes cyclophosphamide, doxorubicin, vincristine, and prednisone [3]. However, relapsed or refractory (R/R) DLBCL presents significant treatment challenges. One of the most recent treatments is CAR T-cell therapy, which has demonstrated highly promising results in several observational studies and clinical trials. The key to the optimal use of CAR T-cell therapy lies in balancing its effectiveness against the side effects associated with it, primarily hematologic toxicity and infections. Additionally, several factors influence the effectiveness of CAR T-cell therapy, including a pro-inflammatory state, the tumor microenvironment, PPM1D mutations, single nucleotide polymorphisms in the cluster of differentiation 19 (CD19) antigens, and circulating monocytes. All these variables should be carefully considered when determining eligibility for treatment.

Compared to the standard of care (SOC) (platinum-based immunochemotherapy), the RCT (transform study), which included 184 patients, demonstrated that lisocabtagene maraleucel (Liso-cel) has higher effectiveness compared to SOC, based on event-free survival and progression-free survival (PFS) (which was double in the Liso-cel group). The study clearly identifies Liso-cel as a preferred second-line treatment for R/R DLBCL over SOC, with a complete response rate of 74% versus 43.5% and a longer duration of response (not reached vs. 9.3 months) [25,26]. An observational study of 95 patients evaluated the negative impact of increased circulating monocytes on response rate and PFS using either Liso-cel or Axi-cel CAR T-cell therapy. Monocyte depletion prior to treatment was associated with a better response. CD86, expressed on monocytes, binds to cytotoxic T-lymphocyte-associated protein 4 (CTLA-4), resulting in lymphocyte activation inhibition. CD163, expressed on monocyte macrophages, could inhibit T-cell activation, leading to poor prognosis. SI6LC5 on monocytes causes CD8+ proliferation impairment and T-cell exhaustion [24].

Although early failure to respond to chemotherapy does not necessarily affect the outcome of CAR T-cell therapy due to its different mechanism of action, a retrospective study of 60 patients with bulky disease (5-10 cm) showed that bulky disease is associated with poor response and outcomes. High levels of lactate dehydrogenase (LDH) and total metabolic tumor volume (TMTV), which correlate with tumor activity and cancer burden, negatively impact response to CAR T-cell therapy and can lead to early relapse after treatment [22]. A retrospective cohort study of 240 patients with R/R DLBCL showed that delayed infusion of Axi-cel, whether due to active infection, disease-related procedures (e.g., thoracocentesis), or logistical causes, results in shorter PFS (1.8 vs. 8.2 months) if the delay was 2-5 days compared to on-time infusion. However, no significant effect was observed if the delay was less than two days (5.7 vs. 8.2 months). Overall survival was also shorter with delays (2-5 days) compared to on-time infusion (6.6 vs. 25.6 months). Patients who experienced delayed infusion had higher levels of CRP but lower levels of absolute neutrophil count (ANC), platelets, and hemoglobin [21].

Half of the global population carries two variants of the germline CD19 antigen, which is the target for CAR T-cell therapy. A single nucleotide polymorphism that encodes leucine or valine at amino acid position 174 of the CD19 antigen can influence treatment response and outcomes [17]. A retrospective observational study of 88 patients demonstrated better clinical outcomes in patients carrying the L174 variant of the CD19 antigen, with a response rate of 51% compared to 30% in CD19 V174 carriers. PFS and overall survival were 22 months and 37 months, respectively, compared to 6 and 8 months in CD19 V174 carriers [17].

A phase II clinical trial involving 21 patients treated with a combination of anti-CD19 and anti-CD20 CAR T-cell therapy reported an objective response rate of 81%, with 52.4% achieving a complete response. Grade 3-4 CRS and neurotoxicity were observed in 28.5% and 9.5% of patients, respectively. The overall survival and PFS were 8.1 months and 5 months, respectively [16]. These results support the use of combination therapy, which yields better clinical outcomes with manageable side effects. In a clinical trial involving 75 patients who underwent lymphodepleting chemotherapy followed by Axi-cel infusion, with an 11-month follow-up, it was found that a pro-inflammatory state and an unfavorable tumor microenvironment are linked to an increased risk of toxicity after CAR T-cell infusion [23]. A baseline elevated IL-6 (≥40 pg/mL) was associated with early onset of CRS and neurotoxicity at one day and four days, respectively. Among these patients, 89% died within 90 days of infusion, and 56% developed grade 3 or higher CRS [23].

A study involving 85 patients with relapsed/refractory DLBCL, with a 20% prevalence of the PPM1D gene mutation, revealed that CRS and immune effector cell-associated neurotoxicity syndrome (ICANS) occurred at similar rates in both mutation-positive and mutation-negative patients. However, clinical outcomes were poorer for those with the PPM1D mutation, with 60% achieving partial remission compared to 56% of the wild-type group who attained complete remission. Furthermore, PFS was 3 months for the mutation group versus 12 months for the wild-type group, and overall survival was 5 months compared to 37 months, respectively [15].

The primary adverse effect of CAR T-cell infusion is hematologic toxicity, which is similar to most standard chemotherapy treatments. We reviewed three studies discussing various aspects of hematologic toxicity associated with this treatment [18-20]. Persistent cytopenia is defined as grade 3-4 cytopenia lasting more than eight weeks [19,20]. Severe cytopenia usually occurs in the first 30 days following CAR T-cell therapy and is most likely associated with lymphodepleting chemotherapy. However, cytopenia can persist beyond three months without evidence of bone marrow dysplasia, indicating that other hematologic toxicities, not related to lymphodepleting chemotherapy, could occur [20]. A non-randomized clinical trial involving 38 patients found that tumor load, CRP, and LDH levels were higher before CAR T-cell therapy in patients with grade 3-4 persistent cytopenia after treatment. Interestingly, PFS and overall survival were higher in patients with grade 3-4 cytopenia than in those without, suggesting that persistent cytopenia following CAR T-cell therapy for R/R DLBCL may indicate higher efficacy but also more adverse events [20].

An observational study involving 31 patients revealed that approximately 58% experienced prolonged hematologic toxicity, which was linked to shorter one-year survival rates compared to those without such toxicity [19]. An observational study of 258 patients found that following CAR T-cell infusion, around 72% of patients experienced profound neutropenia (ANC < 100 cells/mL), and 64% of patients had prolonged neutropenia (≥21 days) [18]. One of the most common side effects of CAR T-cell therapy, in addition to hematologic toxicity, is infection [11-14]. A cohort observational study of 60 patients found that 86.7% of them developed neutropenic fever within the first month. Infections that arise within the first 30 days are typically bacterial, with *Clostridium difficile* identified as the most prevalent pathogen, followed by lobar pneumonia and soft tissue infections. The respiratory syncytial virus was the most common viral infection, followed by cytomegalovirus (CMV) and BK. Infections after 30 days were similarly bacterial predominant, followed by viral infections, half of which were mild. The study indicated that a lower baseline performance status, an ICANS grade of 2 or higher, and exposure to systemic corticosteroids after CAR T-cell therapy were linked to an increased rate of overall infections [13].

Long-term follow-up of patients who have undergone CAR T-cell therapy is very important to understand long-term response and patient outcomes [12]. While many patients achieve complete remission shortly after therapy, the response over the following several years remains the most important factor determining the long-term success of the treatment. Studies have shown that while some patients have PFS for several years, others may develop late relapses, which means that continuous monitoring is essential. Severe side effects such as cytokine release syndrome (CRS) and neurotoxicity raise concerns about the quality of life post-treatment [15]. The impact on post-treatment recovery, cognition, and overall well-being should be considered, highlighting the importance of comprehensive post-treatment care that includes not only physical health but also mental and emotional well-being.

Despite the promising results of CAR T-cell therapy, its high cost is considered a barrier to its widespread adoption. The cost-effectiveness of CAR T-cell therapy is an important issue due to the financial burden it places on patients and healthcare systems. Some strategies to improve accessibility include developing more cost-effective manufacturing processes, increasing the availability of treatment centers, and implementing policies that ensure equitable access to treatment.

The limitations of the systematic review include the fact that few databases were reviewed (PubMed, PMC, Medline, and Cochrane); the exclusion of non-English studies and grey literature might have left out valuable information; the review focused on studies from the past five years; and only published studies were included. Data from different study designs, patient populations, and methodologies could affect the comparability of the findings. Potential biases include selection bias: with a mix of randomized clinical trials and observational studies, there may be differences in patient selection criteria, leading to variability in outcomes. Observational studies are especially prone to this bias. Reporting bias is also a risk, as not all side effects or outcomes may have been reported in the included studies, potentially leading to an incomplete picture of the therapy’s risks.

## Conclusions

In conclusion, CAR T-cell therapy is a highly promising treatment for relapsed or refractory DLBCL, demonstrating greater effectiveness than standard chemotherapy. However, the treatment is associated with several adverse effects, in addition to many factors affecting its effectiveness, which require careful patient selection for this treatment to optimize their clinical outcomes. Future research should continue to focus on how to maximize the benefits of CAR T-cell therapies based on patient characteristics while also minimizing the associated risks. Future research should focus on identifying patient subgroups who benefit the most from CAR T-cell therapy, considering factors like genetic mutations, disease stage, and tumor burden, as well as on standardizing how outcomes and side effects are reported to allow for more reliable comparisons across studies. Additional randomized controlled trials are necessary to validate the findings from observational studies. Research should focus on longer follow-up periods to evaluate the long-term effectiveness and safety of CAR T-cell therapy, particularly regarding survival rates and late-onset adverse effects.
